# Shift from Pro- to Anti-Inflammatory Phase in Pelvic Floor Muscles at Postpartum Matches Histological Signs of Regeneration in Multiparous Rabbits

**DOI:** 10.3390/medicina60040675

**Published:** 2024-04-21

**Authors:** Esteban Rodríguez-Benítez, Kenia López-García, Nicte Xelhuantzi, Dora Luz Corona-Quintanilla, Francisco Castelán, Margarita Martínez-Gómez

**Affiliations:** 1 Centro Tlaxcala de Biología de la Conducta, Universidad Autónoma de Tlaxcala, Tlaxcala 90070, Mexico; stivens.12emsad@gmail.com (E.R.-B.); doraluz.corona.q@uatx.mx (D.L.C.-Q.); fcocastelan@iibiomedicas.unam.mx (F.C.); 2 Doctorado en Ciencias Biológicas, Universidad Autónoma de Tlaxcala, Tlaxcala 90070, Mexico; kenia.lopezg@gmail.com; 3 Facultad de Ciencias de la Salud, Universidad Autónoma de Tlaxcala, Zacatelco 90750, Mexico; xean2806@gmail.com; 4 Departamento de Biología Celular y Fisiología, Instituto de Investigaciones Biomédicas, Universidad Nacional Autónoma de México, Carretera Federal Tlaxcala-Puebla km 1.5 s/n, Tlaxcala 90070, Mexico

**Keywords:** childbirth, histology, inflammation, levator ani, reproduction

## Abstract

*Background and Objectives*: Pelvic floor muscles (PFM) play a core role in defecation and micturition. Weakening of PFM underlies urogynecological disorders such as pelvic organ prolapse and stress urinary incontinence. Vaginal delivery damages PFM. Muscle trauma implies an inflammatory response mediated by myeloid cells, essential for subsequent recovery. Molecular signaling characterizing the pro-inflammatory phase shifts M1 macrophages to M2 macrophages, which modulate muscle repair. The present study aimed to evaluate histological characteristics and the presence of M1 and M2 macrophages in bulbospongiosus (Bsm) and pubococcygeus muscles (Pcm). *Materials and Methods*: Muscles from young nulliparous (N) and multiparous rabbits on postpartum days three (M3) and twenty (M20) were excised and histologically processed to measure the myofiber cross-sectional area (CSA) and count the centralized myonuclei in hematoxylin-eosinstained sections. Using immunohistochemistry, M1 and M2 macrophages were estimated in muscle sections. Kruskal–Wallis or one-way ANOVA testing, followed by post hoc tests, were conducted to identify significant differences (*p* < 0.05). *Results*: The myofiber CSA of both the Bsm and Pcm of the M3 group were more extensive than those of the N and M20 groups. Centralized myonuclei estimated in sections from both muscles of M20 rabbits were higher than those of N rabbits. Such histological outcomes matched significant increases in HLA-DR immunostaining in M3 rabbits with the CD206 immunostaining in muscle sections from M20 rabbits. *Conclusions*: A shift from the pro- to anti-inflammatory phase in the bulbospongiosus and pubococcygeus muscles of multiparous rabbits matches with centralized myonuclei, suggesting the ongoing regeneration of muscles.

## 1. Introduction

Pelvic floor muscles (PFM) play a core role in defecation and micturition. PFM weakening underlies some of the most prevalent and debilitating pelvic floor disorders, such as pelvic organ prolapse (POP) and stress urinary incontinence (SUI). Aging and childbirth are often reported as the main risk factors that weaken PFM. Vaginal birth is considered the leading risk factor, because the passage of the fetal head is associated with exceptional force on, and overstretching, of the PFM [1]. This may lead to their rupture or avulsion [2]. Therefore, childbirth-induced PFM injuries imply neuromuscular and supportive impairments that are prone to the onset of urogynecological disorders like POP or SUI [1,2,3,4,5,6]. In this regard, three-dimensional computer models and magnetic resonance imaging have been able to predict the overstretching of pelvic and perineal muscles, tendons or connective tissue, and nerves [1]. Ultrasonographic data also supports childbirth-related injuries of PFM [2,3,6]. The rate of women diagnosed with puborectalis trauma increases four-fold from pregnancy to the first five days postpartum, matching the enlargement of the levator hiatus [4]. Some of the latter morphometry defects in PFM and functional outcomes coming from neurophysiological outcomes (i.e., evaluation of neuromuscular reflexes and muscle strength) are considered indicators of muscle damage [1,7]. Following metabolic and physical insults, skeletal muscle degeneration and regeneration phases, which comprise orchestrated processes involving injured myofibers, fibroblasts, satellite cells, and infiltrated immune cells, among other cell types [8], lead to muscle recovery [9]. Given that macrophage-driven mechanisms seem to condition muscle regeneration [10], addressing inflammation in PFM is supported by the exploration of cell-based therapies, along with the adequacy of biomaterials to ameliorate pelvic floor dysfunctions [5].

Muscle trauma implies an inflammatory response mediated by myeloid cells essential for subsequent muscle recovery [5]. In the pro-inflammatory phase, neutrophils and M1 macrophages phagocyte cellular debris by expressing molecules like HLA-DR and releasing proteolytic enzymes, oxidative factors, and cytokines [11]. M2 macrophages, including CD-206, express molecules among anti-inflammatory cytokines [11]. Molecular signaling characterizing the pro-inflammatory phase shift of M1 macrophages to M2 macrophages (anti-inflammatory phenotype) modulates muscle repair and could be considered an indicator of muscle regeneration [12].

Whereas inflammation and macrophage influence in the hindlimb muscles have been commonly researched, these mechanisms and influences in PFM have been addressed more recently. In this regard, some PFM of animal species like rodents [13,14,15,16,17,18] and rabbits [19,20,21,22,23] have been used as study models. Simulated birth trauma in rats, comprising the introduction of a catheter balloon into the vagina and its filling to produce vaginal distention, caused morphometry defects, immune cell infiltration, and edema in the external urethral sphincter and levator ani muscles, as well as the pubococcygeus [13,14,18]. Moreover, the leak point pressure, an indicator of a urethral deficit and poor urine continence, became lower, which resembles a model of SUI [13,15]. More recent studies have reported that simulated birth trauma increases levels of IL-6, TNF⍺, and TNFR, suggesting ongoing inflammation in the urethra; exposure to more than one muscle trauma seems to affect them differentially [15]. Findings from rats support that adverse outcomes of vaginal distention depend on the evaluated pelvic floor, which may be associated with the plastic adaption of each individual during pregnancy and postpartum [16].

The female rabbit is another well-suited organism to evaluate the anatomical organization and function of the pelvic floor and its PFM in reproductive contexts for biomedical interests [19,20,21,22,23]. Our workgroup has focused on the bulbospongiosus (Bsm) and pubococcygeus muscles (Pcm), given their contribution to urine storage and voiding [19]. Findings from the Bsm and Pcm of pregnant, primiparous, and multiparous rabbits suggest that their differences in damage and recovery may underlie PFM functional plasticity at postpartum [21,23]. In addressing representative markers for muscle degeneration and regeneration at postpartum, we have reported elsewhere that muscle regeneration processes seem to recover Bsm faster than the Pcm in multiparous rabbits [21]. Accordingly, we hypothesize that the M2 macrophages of the Bsm increase on day 20 postpartum, while the M1 macrophages increase for the Pcm in the same time frame. Overall, the present study aimed to evaluate the histological characteristics of, and the presence of M1 and M2 macrophages in, the Bsm and Pcm of young nulliparous and multiparous rabbits on postpartum days three and twenty.

## 2. Materials and Methods

### 2.1. Animals

Eighteen young chinchilla-breed female rabbits (*Oryctolagus cuniculus*) were housed in individual stainless-steel cages (50 × 60 × 40 cm) and kept at 22 ± 2 °C under artificial lighting conditions (L:D 16:8; lights on at 0600 h) in the vivarium of the Centro Tlaxcala de Biología de la Conducta (Universidad Autónoma de Tlaxcala). All rabbits had daily free access to pellet food and tap water. The procedures below followed the guidelines of, and were approved (Protocol ID 6310 approved on 25 July 2019) by, the Ethics Committee of the Instituto de Investigaciones Biomédicas-Universidad Nacional Autónoma de México.

We allocated the rabbits into three groups: nulliparas (n = 6), and multiparas euthanized on postpartum day 3 (n = 6) or day 20 (n = 6). Multiparous rabbits had their first mating at six months of age; henceforth, rabbits mated again during the first postpartum days after the first, the second, and the third delivery [21]. After the fourth delivery, the pups were euthanized to allow multiparous rabbits a hormonal status more similar to nulliparous rabbits [21].

Age-matched nulliparas and multiparas were euthanized with an overdose of sodium pentobarbital (120 mg/kg; i.p.). Next, animals were placed supine to harvest the Bsm and Pcm, and transferred into Bouin-Duboscq fixative for 24 h, as explained elsewhere [23]. After fixation, muscles were dehydrated in ascending ethanol concentrations (70, 80, 96, and 100%), cleared in xylene, and embedded in Paraplast X-tra (Sigma-Aldrich, St. Louis, MO, USA). We obtained 7 µm thickness traverse muscle sections with a microtome (RM2135, Leica, Wetzlar, Germany) and placed them serially on poly-L-lysine coated slides. Next, some sections were stained with hematoxylin and eosin (HE), while others were used for immunohistochemistry (see below).

### 2.2. Histology

**Figure 1:**
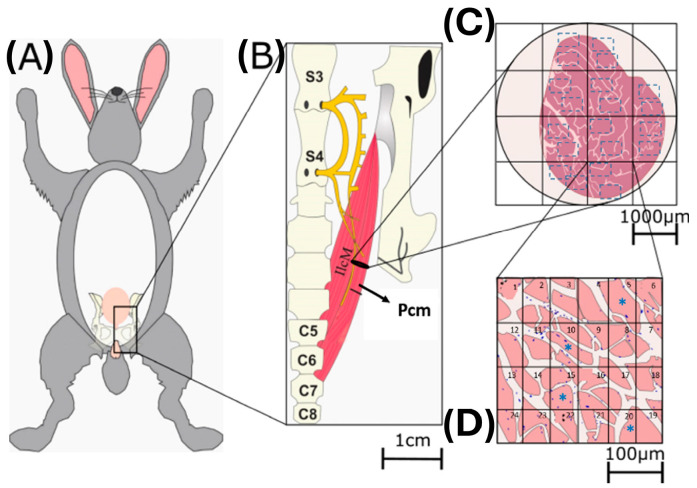
Sampling PFM myofibers. The female rabbit was placed in a supine position (**A**), and the Bsm and Pcm muscles were identified and excised (**B**); subsequently, they were histologically processed and cut at seven μm, and a section was taken from the medial region and stained. HE, photos were taken in the microscope at 50× to reconstruct the entire muscle, and the results were divided into 16 quadrants. Of said reconstruction of the muscle, two photos were taken at 40× for each quadrant (**C**). Each photo at 40× was divided into 24 quadrants, and each beech fiber was measured in the fifth quadrant (**D**). Blue asterisks indicate sampled myofibers.

We measured the cross-sectional area and myonuclei of 50 myofibers from the medial regions of the Bsm and Pcm [23]. For this sake, photographs were taken at 50×, and the entire muscle cross-section was reconstructed. The reconstruction was divided into sixteen quadrants to take two photos at 40× in each quadrant with an OLYMPUS camera (Tokyo, Japan) connected to a visible light microscope (Nikon ECLIPSE E600, Tokyo, Japan). Each field, in turn, was divided into twenty-four quadrants with the support of a grid, and the Axio Vision Rel. 4.6 (Carl Zeiss AG, Oberkochen, Germany) program was used to measure the myofiber cross-sectional area (CSA) of one fiber located in every fifth quadrant (Figure 1). Centralized and internalized myonuclei were manually counted in the sampled myofibers by one observer (ERB) that was blinded to the slide ID. The resulting data were averaged (per muscle per animal) and represented as the percentage of myonuclei per myofiber.

### 2.3. Immunohistochemistry

For addressing the inflammatory response in both the Pcm and Bsm, muscle sections were immunostained with HLA-DR (ab166777, Abcam, Cambridge, UK) or anti-CD206 antibodies (MCA2155, Bio-Rad, Hercules, CA, USA) to identify M1 or M2 macrophages. We adapted a protocol reported elsewhere [23]. Briefly, muscle sections were deparaffinized before retrieving antigens by incubating them with sodium citrate (pH 6). Sections were incubated in an H_2_O_2_ solution at room temperature for 30 min to quench endogenous peroxidases before blocking non-specific sites with 5% normal goat serum (Santa Cruz Biotechnology, Dallas, TX, USA) diluted in PBS for one hour. The sections were washed with PBS-triton solution before incubation with anti-HLA-DR (1:200) and anti-CD206 (1:200) overnight at 4 °C. Spleen sections from nulliparous rabbits were used as the positive control; negative controls consisted of sections where the primary antibody was omitted. Sections were washed with PBS-triton x-100 and incubated with biotinylated goat polyclonal anti-mouse IgG antibodies (1:250, sc-2309, Santa Cruz Biotechnology). We used the Vectastain ABC kit (Vector Labs, Newark, CA, USA) to develop the immunostaining. Sections were counter-stained with Mayer’s hematoxylin, dehydrated in ascending concentrations of ethanol cleared with xylene, and mounted with mounting medium (entellan). Photographs were taken with an OLYMPUS camera connected to an optical microscope (Nikon ECLIPSE E600). For counting M1 and M2 macrophages, the entire muscle was reconstructed, followed by drawing a grid (3 × 4 quadrants) to sample alternate quadrants. Representative photographs were taken using a Ni-NU microscope (Nikon) coupled to a digital camera.

### 2.4. Statistical Analyses

The normality of data was assessed with Kolmogorov–Smirnov tests. Subsequently, we used Kruskal–Wallis followed by Dunn’s multiple comparisons tests, or one-way ANOVA followed by Tukey tests, to determine significant differences (*p* < 0.05) among the groups. Data are shown as median (minimum to maximum value) or mean ± S.E.M. unless otherwise stated. All the analyses were performed using the Prism 9 (GraphPad, Boston, MA, USA) program.

## 3. Results

**Figure 2:**
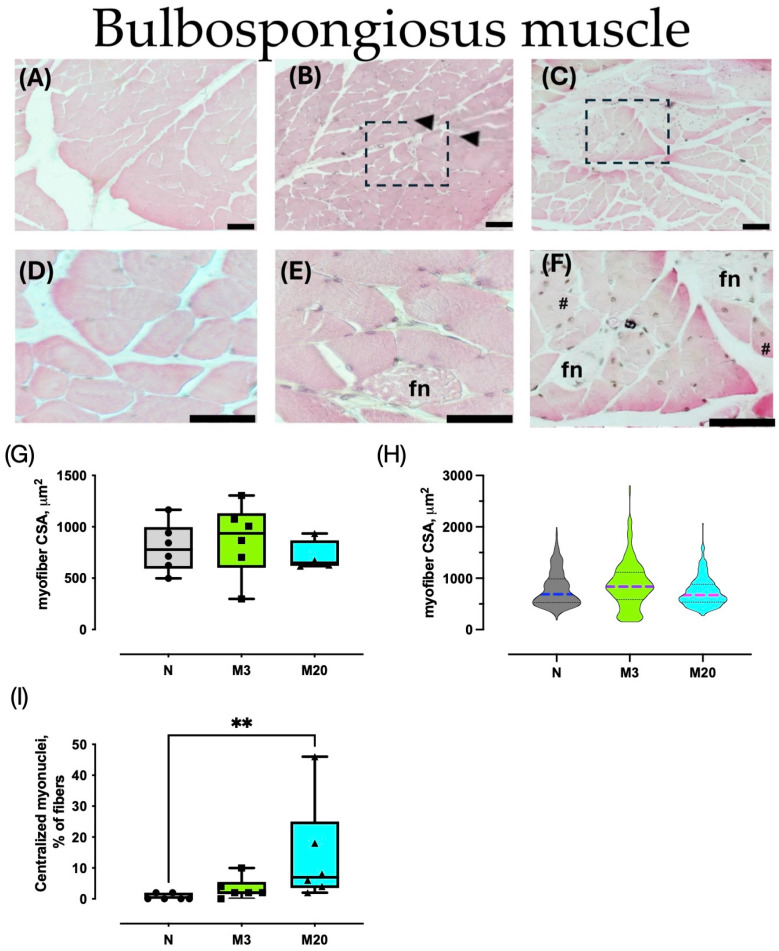
Morphometry of bulbospongiosus (Bsm) muscles of nulliparous (N) and multiparous rabbits at postpartum days 3 (M3) and 20 (M20). Representative images of hematoxylin-stained sections (**A**–**F**). Data are mean ± S.E.M., (**G**) or median and interquartile range (**I**). Violin plots represent the data of 300 myofibers (50 per animal) per group (n = 6 rabbits each) (**H**). Arrowhead, hypercontracted myofibers; fn, focal necrosis; dashed rectangle, PMN cells; #, internalized, and centralized myonuclei. Bar, 50 µm ((**A**–**C**) 100×; (**D**–**F**), 400×). **, *p* < 0.01.

**Figure 3:**
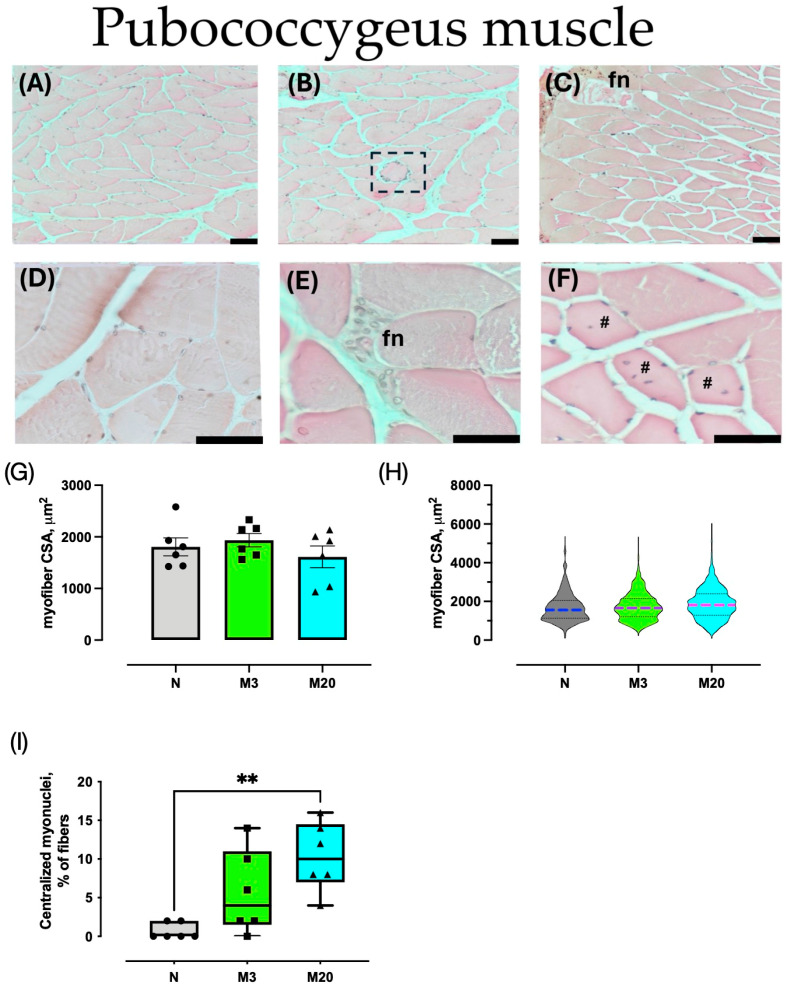
Morphometry of pubococcygeus (Pcm) muscles of nulliparous (N) and multiparous rabbits at postpartum days 3 (M3) and 20 (M20). Representative images of hematoxylin-stained sections (**A**–**F**). Data are mean ± S.E.M., (**G**) or median and interquartile range (**I**). Violin plots represent the data of 300 myofibers (50 per animal) per group (n = 6 rabbits each) (**H**). Representative images are shown. Arrowhead, hypercontracted myofibers; fn, focal necrosis; dashed rectangle, PMN cells; #, internalized, and centralized myonuclei. Bar, 50 µm ((**A**–**C**) 100×; (**D**–**F**), 400×). **, *p* < 0.01.

Histological characteristics of the Bsm and Pcm were observed in H–E sections (Figure 2 and Figure 3). For both muscles, nulliparous rabbits showed typical polygonal myofibers with peripheral nuclei, compacted by a well-delimited endo- and perimysium (Figure 2A,D and Figure 3A,D). In high contrast, muscle sections from multiparas (M3 and M20 groups) exhibited signs of histopathological damage, including centralized and internalized myonuclei, focal necrosis, hyper-contracted fibers, and PMN cells (Figure 2B,C,E and Figure 3B,C,E).

### 3.1. Muscle Morphometry

We analyzed HE-stained sections to measure the myofiber cross-sectional area and count the peripheral and central myonuclei. The average Bsm myofiber CSA did not vary significantly among the groups (Kruskal–Wallis = 1.636, *p* = 0.4699; Figure 2G); the same was true for the Pcm (F = 0.865, *p* = 0.441; Figure 3G). Further analyses of the CSA distribution supported the latter findings (Figure 2H and Figure 3H).

We further estimated the ratio of sampled myofibers having centralized myonuclei (as a percentage) as a histological indicator of muscle regeneration. The Bsm indicated significant differences among the groups (Kruskal–Wallis = 9.732, *p* = 0.0028), and post hoc tests indicated a significant augmentation in the M20 vs. N group (Figure 2I). Similarly, the percentage of Pcm myofibers with centralized myonuclei varied between nulliparas and multiparas (Kruskal–Wallis = 10.05, *p* = 0.0024), which was prompted by the significant increase measured in the M20 compared to the N group (*p* = 0.0047; Figure 3I).

### 3.2. HLA-DR Immunostaining

**Figure 4:**
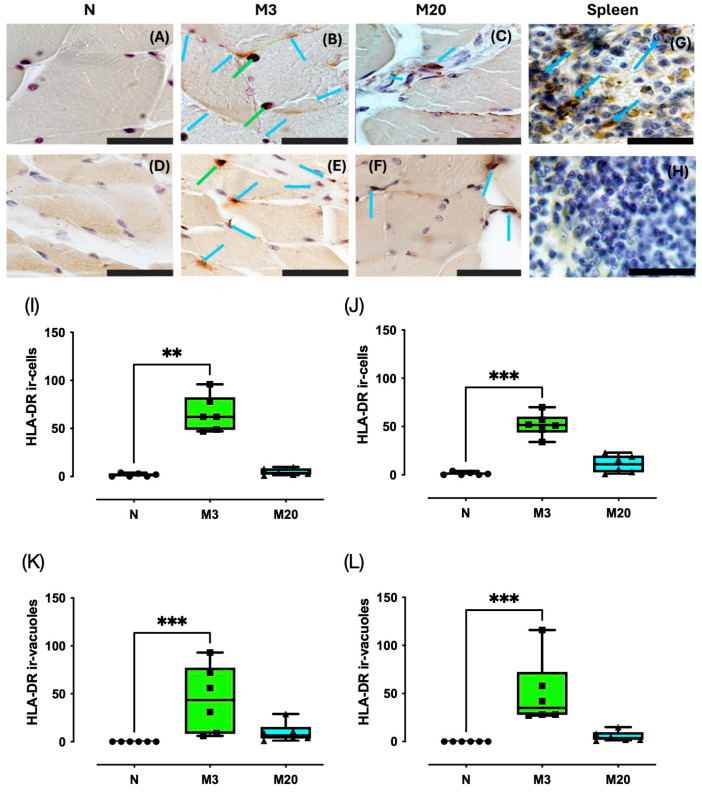
HLA-DR immunostaining in bulbospongiosus (Bsm); (**A**–**C**,**I**,**J**) and pubococcygeus (Pcm); (**D**–**F**,**K**,**L**) muscles of nulliparous (N) and multiparous (M) rabbits. Spleen sections from nulliparous rabbits were used as a positive control (**G**); the negative control was established using spleen sections in which the primary antibody was omitted (**H**). Data are median and interquartile range. **, *p* < 0.01; ***, *p* < 0.001. Blue arrows, HLA-DR-ir cells; green arrows, HLA-DR-ir-vacuoles Bar, 100 µm (600×).

We carried out anti-HLA-DR immunohistochemistry in the Bsm and Pcm of nulliparous and multiparous rabbits (Figure 4A–F). Spleen sections were used as positive controls; non-specific staining was assessed by incubating slides without the primary antibody (Figure 4G,H). The HLA-DR immunostaining was observed in the cytoplasm of resembling PMN cells and in strongly stained vacuole patterns (Figure 4B,E). Therefore, we estimated the number of HLA-DR ir-cells and HLA-DR ir-vacuoles.

For the Bsm, we found that HLA-DR ir-cells changed significantly among the groups (Kruskal–Wallis = 12.76, *p* < 0.0001), prompted by a significant increase detected in comparing the M3 vs. N groups (*p* = 0.0014) (Figure 4I). On the other hand, there were no significant (*p* > 0.05) variations between the N vs. M20 (*p* = 0.7625) and the M3 vs. M20 groups (*p* = 0.0545). Such findings matched well with those for the Pcm, particularly the significant differences among the nulliparas and multiparas (Kruskal–Wallis = 13.90, *p* < 0.0001; Figure 4J) and the significant increase (*p* < 0.0006) of the HLA-DR ir-cells of the M3 vs. N group. No significant differences were detected when comparing the N vs. M20 (*p* = 0.7625) and M3 vs. M20 groups (*p* = 0.0545).

Statistical tests indicated significant differences regarding the estimated number of HLA-DR ir-vacuoles for the Bsm (Kruskal–Wallis = 13.72, *p* < 0.0001; Figure 4K) and Pcm sections among the groups (Kruskal–Wallis = 15.76, *p* < 0.0001; Figure 4L). Post hoc tests indicated a significant increase in the HLA-DR-ir-vacuoles for the M3 vs. N group for both the Bsm (*p* = 0.0007) and Pcm (*p* = 0.0002).

### 3.3. CD206 Immunostaining

**Figure 5:**
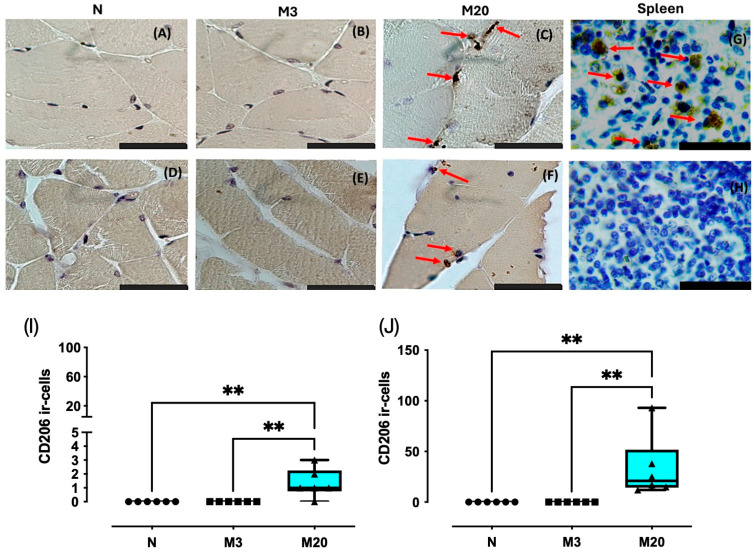
CD206 immunostaining in bulbospongiosus (Bsm); (**A**–**C**,**I**) and pubococcygeus (Pcm); (**D**–**F**,**J**) muscles of nulliparous (N) and multiparous (M) rabbits. Spleen sections from nulliparous rabbits were used as the positive control (**G**); the negative control was established using spleen sections in which the primary antibody was omitted (**H**). Data are median and interquartile range. **, *p* < 0.01. Red arrows, CD206-ir cells. Bar, 100 µm (600×).

We observed cytoplasmic CD206 immunostaining in Bsm and Pcm, consistent with CD206 ir-cells observed in spleen sections (positive control); no staining was seen when the primary antibody was omitted (Figure 5). Remarkably, no CD206 ir-cells were detected in muscle sections from the N and M20 groups (Figure 5A,B,D,E). The number of CD206 ir-cells for the Bsm and Pcm changed significantly among the groups (Bsm: Kruskal–Wallis = 12.73, *p* = 0.0021; Pcm: Kruskal–Wallis = 16.13, *p* = 0.0002). Post hoc tests indicated that the number of CD206 ir-cells for the Bsm of M20 rabbits was significantly higher than both the N and M3 rabbits (*p* = 0.006 for both pairwise comparisons) and Pcm (*p* = 0.0015 for both comparisons) (Figure 5I,J).

## 4. Discussion

Labor trauma impairs connective tissues and muscles, leading to the onset of pelvic floor disorders. Some cases of SUI are transient, while others are long-lasting. Pelvic organ prolapse complications may imply the need for surgical procedures. Therapies for both SUI and POP temporarily improve some of the pathological signs, which could also be due to factors related to menopause and aging, among others. Inflammation may also influence PFM recovery. The present findings demonstrate that centralized myonuclei in both the Bsm and Pcm increase by twenty days postpartum, matching significant increases in the HLA-DR immunoreactive cells (M1 macrophages) on day three postpartum and in the CD206 immunoreactive cells (M2 macrophages) on day twenty postpartum.

We have reported that multiparity increases the Pcm myofiber CSA without modifying both variables in the Bsm [24]. Such results agree well with the Bsm, but not with the Pcm, for which higher CSA has been reported elsewhere [24]. Similar to the findings herein, the average myofiber CSA for neither Bsm nor Pcm changed among nulliparous, late-pregnant, and primiparous rabbits in a previous study [23]. The discrepancy between multiparas findings may rely on the different regions of muscle that were sampled. In rats, simulation of birth trauma affected the histology of the coccyges and pubococcygeus muscles in the enthesis of each [25,26]. In addition, the damage caused by eccentric exercise, which is expected during vaginal delivery, has been found to be increased in the proximal region of hindlimb muscles such as the rectus femoris [25,27].

Present data, and other data reported elsewhere, have been obtained from the medial regions of Bsm and Icm, where the content of myofibers is predominant [24]. Therefore, signs of muscle injury like focal necrosis, hypercontractile myofibers, and PMN cell infiltration on postpartum days 3 and 20 could provide information about ongoing inflammation. In this regard, the findings herein show consistency with previous reports from rat studies [13]. The infiltration of PMN cells in the Bsm and Pcm agrees with the histological observations and biochemical indicators of muscle damage (e.g., β-glucuronidase activity) reported for late-pregnant, primiparous, and multiparous rabbits [21,23]. Remarkably, histological modifications reported for rabbits subject to reproductive challenges seem mild compared to those of rats subject to multiple simulated birth traumas, lacking the hormonal milieu surrounding the delivery [11,15]. The latter could be explained in terms of PFM adaptions occurring at the end of pregnancy, as reported in rats [16]. Overall, data from histological analyses suggest have suggested that childbirth-induced muscle damage is asymmetrical among myofibers, which may influence the further development of therapies based on neurostimulation or biomaterials [25,27,28].

We used anti-HLA-DR and -CD206 as reliable markers of M1 and M2 macrophages, respectively [11,29]. HLA-DR M1 macrophages and/or mononuclear cell infiltration matches pro-inflammatory responses in striated muscles. TNF-alpha up-regulates the HLA-DR expression in IFN-gamma-treated myoblasts, which may signal autophagy-mediated antigen presentation [30]. Furthermore, HLA-DR expressing T-helper cells could also be present in infiltrates appearing after exercise-related muscle injury [31]. We observed HLA-DR immunostaining in cells, easily seen at 600×, along with giant vacuoles observed at 100× magnification, given the association between low muscle strength and inflammatory cells in the biceps brachii of people with polymyositis and dermatomyositis [29,30,32]. Our present data provide supporting evidence of ongoing inflammation in both the Bsm and Pcm on postpartum day three. Such an increase was not observed in the muscles of multiparas on postpartum day 20.

Conversely to HLA-DR immunostaining, that of CD206 was almost absent in nulliparous and M3 rabbits. M2 macrophages attenuate the M1 macrophages and secrete molecules that enhance muscle recovery, including IL-10, TGFβ, and miR-501 [9,11,17]. Indeed, the latter observation matched a significant increase in the centralized myonuclei and the estimated number of M2 macrophages (CD206 positive) for the Pcm, suggesting active muscle regeneration in agreement with the expression of muscle regeneration markers such as MyoD, MyoG, and desmin [21]. In contrast, CD206 positive M2 macrophages measured on postpartum day 20 increased to a lesser extent in the Bsm than the Pcm, likely indicating that muscle regeneration occurred faster than in Pcm [21]. Thus, a single delivery or multiple deliveries may trigger the kinetics of the inflammatory response in the PFM (through the presence of M1 and M2 macrophages), as a fundamental part of muscle injury and efficient regeneration. Such a notion agrees with the significant increase in myofibers, showing centralized myonuclei in the Bsm and Pcm, in multiparas on postpartum day 20.

Pelvic floor muscle injuries during childbirth often lead to disorders that impair the quality of life for women [1]. Muscle damage implies macrophage and satellite cell interactions, among other cell types that regulate degeneration and regeneration processes in PFM [9]. The latter interactions have underlined some targets of interest in developing therapies for ameliorating deleterious urogynecological symptoms. In female rats, the administration of anti-inflammatory drugs has been found to impair PFM recovery after simulating birth injury to PFM [18], which is likely associated with the roles of muscle M1 and M2 detected 3 and 7 days post-injury [33]. Indeed, M2 macrophages make an important contribution to the muscle regeneration process by interacting with muscle satellite cells through anti-inflammatory cytokines and other molecules such as miR-501 [9,10,12,17]. A recent study has reported that a pro-regenerative extracellular matrix hydrogel can modulate the immune response, myogenesis, and extracellular matrix remodeling, thus exerting a protective effect on PFM after simulated birth injury [33]. Indeed, injury is different among individual PFM [27]. In this context, our present findings extend the challenge of understanding PFM disorders, to evaluate the transition from M1 to M2 macrophages in the PFM of multiparous rabbits that contribute differentially to either urine voiding (e.g., the Bsm) or continence (e.g., the Pcm) [19]. In addition to being the first report in which pro- and anti-inflammatory states are evaluated at postpartum in a physiological model like multiparity, our findings could boost further studies on the plasticity of immune responses, along the reproductive experience of females.

Limitations of the present work include the lack of investigation of molecules involved in muscle injury or regeneration (e.g., TNF⍺, TGF-β) and mature myosin isoforms that prove informative in relation to the extent of functional recovery. In contrast, one of the remarkable strengths of this study was represented in its addressing of pro- and anti-inflammatory processes in an animal model subject to reproductive challenges that imply adjustments in hormone actions, which are expected to occur in women postpartum.

## 5. Conclusions

A shift from the pro- to anti-inflammatory phase in the bulbospongiosus and pubococcygeus muscles of multiparous rabbits matches with centralized myonuclei, suggesting ongoing regeneration of the bulbospongiosus and pubococcygeus muscles.

## Data Availability

The raw data supporting the conclusions of this article will be made available by the authors upon request.
